# Increased prelaminar tissue thickness in patients with open-angle glaucoma and type 2 diabetes

**DOI:** 10.1371/journal.pone.0211641

**Published:** 2019-02-07

**Authors:** Yoon Seop Sim, Jin-Woo Kwon, Donghyun Jee, Jin A. Choi, Seung-Hyun Ko, Chan Kee Park

**Affiliations:** 1 Department of Ophthalmology and Visual Science, St. Vincent's Hospital, College of Medicine, The Catholic University of Korea, Seoul, Republic of Korea; 2 Division of Endocrinology and Metabolism, Department of Internal Medicine, St. Vincent Hospital, College of Medicine, Catholic University of Korea, Seoul, Republic of Korea; 3 Department of Ophthalmology and Visual Science, Seoul St. Mary's Hospital, College of Medicine, The Catholic University of Korea, Seoul, Republic of Korea; The University of Melbourne, AUSTRALIA

## Abstract

**Background:**

The characteristics of the optic nerve head (ONH) in open angle glaucoma (OAG) patients with diabetes have not been reported. This study aimed to characterize the ONH structures and glaucomatous damage in diabetic OAG patients, using age-matched non-diabetic OAG patients and control subjects.

**Methods:**

The locations of visual field defects of OAG patients were classified and the prelaminar thickness and lamina cribrosa depth were measured in 64 OAG patients with type 2 diabetes (OAG+DM), 68 OAG patients without diabetes (OAG-DM), and 36 controls. All participants were scanned by spectral domain-optical coherence tomography. The anterior prelaminar depth and lamina cribrosa depth were measured at the center of the reference line (the Bruch’s membrane opening plane). The prelaminar tissue thickness was obtained by subtracting the anterior prelaminar depth from the anterior lamina cribrosa depth.

**Results:**

The visual field defects in the OAG+DM group were more commonly found in the inferior hemifield (*P* = 0.010), and tended to involve the central visual field compared to the OAG-DM group (*P* = 0.044). In the comparison of ONH parameters, the prelaminar thickness was highest in the OAG+DM group, followed by the control subjects and the OAG-DM group (*P* = 0.035). *Post-hoc* testing showed that prelaminar thickness was significantly greater in the OAG+DM group than in the OAG-DM group (*P* = 0.033). The lamina cribrosa depth was deepest in the OAG+DM group, followed by the OAG-DM group and the control subjects (*P* = 0.006).

**Conclusions:**

Diabetic and non-diabetic OAG patients exhibit different characteristics of glaucoma, particularly increased prelaminar thickening in diabetics.

## Introduction

Diabetes is associated with many ocular complications. Although diabetic retinopathy (DR) is the most well-known complication of diabetes, patients with diabetes may have other ocular complications such as cataract, corneal disease, glaucoma and optic disc abnormalities such as anterior ischemic optic neuropathy, and diabetic papillopathy.[1]

In recently published meta-analyses, diabetes increased the prevalence of glaucoma with a relative risk of 1.48.[2] Neurovascular coupling is impaired in the early stages of DR, and neurodegeneration of the optic nerve occurs even before the onset of clinically detectable DR.[3, 4] However, except for neovascular glaucoma caused directly by diabetes, the relationship between diabetes and glaucoma is still not completely known. The Rotterdam Eye Study reported that the presence of diabetes was not associated with open-angle glaucoma (OAG).[5] The ocular hypertension treatment study reached a similar conclusion, with the presence of diabetes protecting against the development of OAG, with a hazard ratio of 0.40 (0.18–0.92).[6]

However, diabetes is significantly associated with increased intraocular pressure (IOP).[2, 7] In recently published meta-analyses, the presence of diabetes was associated with an increase of IOP of 0.18 mmHg, and with an increase in 10 mg/dl in fasting glucose was 0.09 mmHg.[2] Although the association between diabetes and IOP is weak, the results are consistent throughout the population–based studies. A high glucose level in aqueous humor of patients with diabetes may accelerate the depletion of trabecular meshwork cells by accumulation of fibronectin in trabecular meshwork.[8] The aqueous level of transforming growth factor-β2 is particularly high in glaucoma patients with diabetes, compared to those without it.[9, 10]

In subjects with diabetes, functional changes and thinning of the inner retina due to neural degeneration have been reported, even before clinically visible retinal changes occur.[11, 12] And this inner retina thinning occurs especially on the superior side of the optic nerve head (ONH), which is clearly different from those resulting from glaucomatous RNFL damage, which occurs predominantly in the inferior temporal side of ONH.[13, 14] In addition, biomechanical properties of the ONH are affected by diabetes, including increased stiffness,[15] and advanced glycation end products accumulate in the ONH in diabetics.[16] These findings suggest that the ONH may exhibit different characteristics between diabetic and non-diabetic patients. However, the characteristics of the ONH in OAG patients with diabetes have not been reported. In the present study, we characterized the structure of the ONH and glaucomatous damage in type 2 diabetic OAG patients, using age-matched non-diabetic OAG patients and control subjects. Our research focused only on subjects with type 2 diabetes, because type 1 and 2 diabetes have a difference in pathogenesis.

## Materials and methods

### Study subjects

In this cross-sectional retrospective study, OAG patients with type 2 diabetes (OAG+DM), age-matched OAG patients without diabetes (OAG-DM), and non-diabetic, non-glaucomatous controls, all of whom had visited the Glaucoma Clinic of St. Vincent’s Hospital at Catholic University of Korea, and underwent enhanced depth imaging spectral-domain optical coherence tomography (OCT) of the optic nerve head between July 2014 and July 2015, were included. The study was conducted in accordance with the ethical standards of the Declaration of Helsinki and was approved by the Institutional Review Board of St. Vincent’s Hospital, the Catholic university, College of Medicine (VC14RISI0153), which waived the written informed consent because of the study’s retrospective design.

Study subjects underwent a review of their medical history and a full ophthalmic evaluation including the following: a best-corrected visual acuity measurement, slit-lamp biomicroscopy, gonioscopy, Goldmann applanation tonometry, a dilated fundus examination, optic disc and red-free retinal nerve fiber layer (RNFL) photography using a digital fundus camera (CF-60UD; Canon, Tokyo, Japan), Cirrus HD- OCT (Carl Zeiss Meditec, Dublin, CA, USA), and standard automated perimetry 24–2 (Swedish Interactive Threshold Algorithm standard program, Humphrey Visual Field Analyzer; Carl Zeiss Meditec). Patients with at least 2 years of follow-up comprising more than three visual field tests were chosen.

All subjects were required to have a BCVA of 20/40 or better, a with a spherical refraction of -8.0 to +3.0 diopters (D), cylinder correction within ±3.0 D, and clear ocular media, and a normal anterior chamber angle. Participants with the following were excluded: neurological diseases that could affect the visual field (VF) results, a history of ocular surgery other than cataract extraction, or eyes yielding consistently unreliable VF results (defined as >25% false-negative results, >25% false-positive results, or >20% fixation losses).

Glaucoma was defined by the presence of glaucomatous optic neuropathy associated with typical reproducible VF defects evident on standard automated perimetry. A glaucomatous VF change was defined as a glaucoma hemifield test result outside normal limits and the presence of at least three contiguous points in the pattern deviation plot with values of P < 5%, with at least one point associated with a value of P < 1% (excluding points directly above or below the blind spot), on two consecutive reliable standard automated perimetry examinations.

Diabetes was diagnosed in subjects with a fasting plasma glucose ≥ 126 mg/dL or with symptoms of diabetes plus casual plasma glucose concentrations of ≥ 200 mg/dL, based on the 1997 and 2003 revisions of the American Diabetes Association guidelines.[17] DR was graded by retinal specialists (D.-H.J. and J.-W.K) assigned to each eye according to the modified Airlie House classification system. DR, if present, was categorized into five categories, following the international clinical DR severity scales: non-diabetic retinopathy (equivalent to the Early Treatment Diabetic Retinopathy Study [ETDRS] scale level 10), mild non-proliferative diabetic retinopathy (NPDR; equivalent to ETDRS 20), moderate NPDR (equivalent to ETDRS 35, 43, and 47), severe NPDR (equivalent to ETDRS 53A–53E), and proliferative diabetic retinopathy (PDR) (equivalent to ETDRS ≥ 61).[18] We excluded eyes with type 1 diabetes, PDR or neovascular glaucoma, the presence of diabetic macular edema, or any other retinal disease. Patients with interventions such as panretinal photocoagulation and intravitreal injection were also excluded.

The control group had a normal optic disc appearance upon examination of color stereoscopic photographs (an intact neuroretinal rim without peripapillary hemorrhage, thinning, or localized pallor), an IOP ≤ 21 mmHg, the absence of any RNFL abnormality visible on red-free fundus photographs, and normal VF test results. A normal VF presentation was defined as a glaucoma hemifield test result within normal limits, with mean and pattern standard deviation values associated with probabilities of normality > 5%.

### Classification of VF defects

The VF defects of OAG patients were classified according to the pattern standard deviation, as described by Keltner *et al*.[19] First, the defects were classified into three groups involving the superior, inferior, or both hemifields. Then they were classified into two groups including patients with and without involvement of the central 10° of the VF.[20, 21] The VF defect was classified independently in random order and in a blinded manner, without knowledge of the clinical information, by 2 of the authors (J,-A.C., and Y,-S.S).

### Optical coherence tomography protocols and analyses

**Fig 1:**
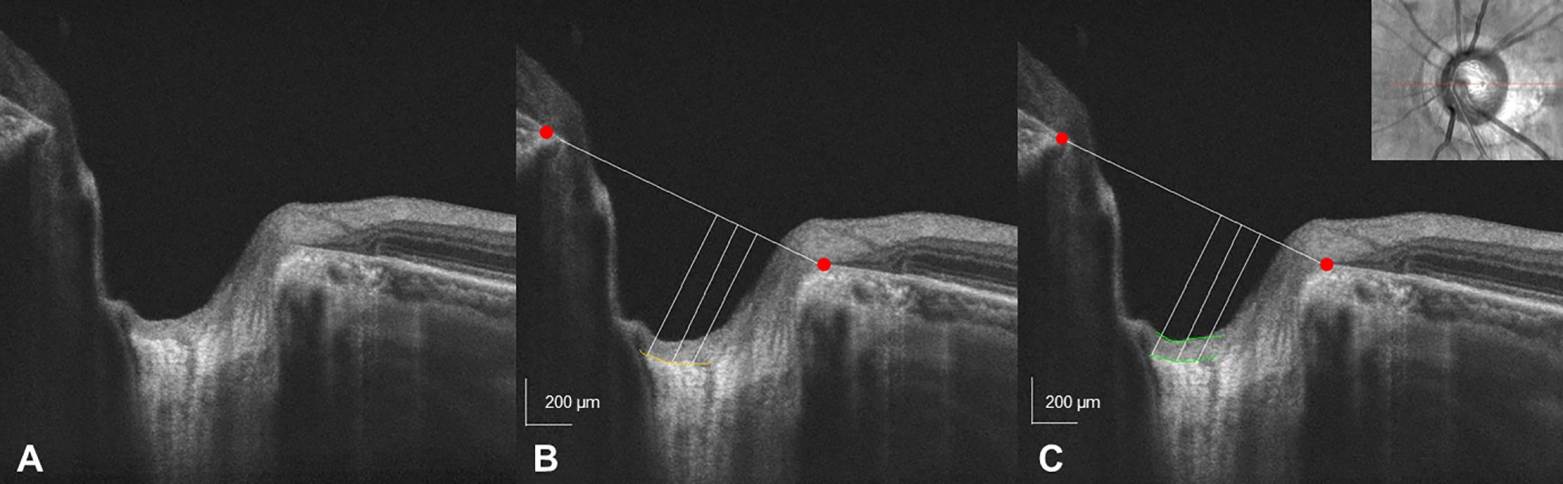
An optical coherence tomography optic nerve head image for measurement of the anterior lamina depth and prelaminar thickness. At the center of the optic nerve head, the reference line (*white horizontal line*) connects the Bruch’s membrane opening (indicated as *red dots*). From a perpendicular line drawn from the reference line, the distance to the level of the anterior surface of the prelaminar tissue and lamina cribrosa were measured at three points: the maximally depressed point and two additional points (100 μm and 200 μm from the maximally depressed point in the temporal direction). For each patient, the mean of three values was used. The anterior lamina cribrosa depth was defined as the distance from the Bruch’s membrane opening plane to the level of anterior surface of the lamina cribrosa (*yellow line*) (B). The anterior prelaminar depth was determined by measuring the distance from the Bruch’s membrane opening plane to the level of the anterior surface of the prelaminar tissue, and the prelaminar tissue thickness was obtained by subtracting the anterior prelaminar depth from the lamina cribrosa depth (gre*en line*) (C).

Imaging with spectral domain optical coherence tomography (SD-OCT) (Cirrus high-definition OCT, Carl Zeiss Meditec) was performed after ≥6mm pupil dilation with a 0.5% solution of phenylephrine hydrochloride and 0.5% tropicamide. The Cirrus SD-OCT automatically detected the center of the disc and then drew a circumpapillary circle from the cube dataset for RNFL thickness analyses, using the built-in analysis algorithm. The average and clock-hour peripapillary RNFL thicknesses and the ONH parameters (disc area, rim area, and vertical cup-to disc ratio) were recorded. In addition, a horizontal section running through the center of the ONH and fovea was scanned using a 1 HD line scan (9 mm length). The HD line scans were performed in enhanced depth imaging mode, as described in a previous study.[22] All measurements were performed using Image J software (National Institutes of Health, Bethesda, Maryland, USA). The anterior prelaminar depth was determined by measuring the distance from the Bruch’s membrane opening plane to the level of the anterior surface of the prelaminar tissue, and the anterior lamina cribrosa depth was defined as the distance from the Bruch’s membrane opening plane to the level of anterior surface of the lamina cribrosa (Fig 1). The distance from the reference line to the level of the anterior surface of the prelaminar tissue and anterior and posterior surface of the lamina cribrosa were measured at three points: the maximally depressed point and two additional points (100 μm and 200 μm from the maximally depressed point in the temporal direction).[23, 24] For each patient, the mean of three values was used. The prelaminar tissue thickness was obtained by subtracting the anterior prelaminar depth from the anterior lamina cribrosa depth (Fig 1C). In 31 of the 168 patients (18.4%), the posterior borders of the lamina cribrosa were not clear enough to be identified.

Subfoveal choroidal thickness was defined as the vertical distance from the hyperreflective line of Bruch’s membrane to the hyperreflective line of the inner surface of the sclera.[25] The thicknesses of the choroid at regions 1.5 mm nasal and temporal to the fovea were also examined, and the average value of three measurements was defined as the average macula choroidal thickness. All images were reviewed by two of the investigators (YSS, CJA). To evaluate the interobserver reproducibility of our measuring method, 15 randomly selected SD-OCT datasets were evaluated by 2 investigators and the intraclass correlation coefficient (ICC) was calculated. Images with a signal strength < 6, obvious motion or blinking artifacts, or poor-quality enhanced depth imaging OCT images were excluded.

### Data analyses

The data were analyzed using SPSS statistical software for Windows, version 16 (SPSS, Chicago, IL, USA). The data were tested for normal distribution using the Shapiro-Wilk test. One-way analysis of variance with a Bonferroni *post hoc* test was used to compare the clinical characteristics of OAG patients with or without type 2 diabetes and control subjects. Categorical parameters in baseline characteristics and the distribution of VF defects in each group were compared using the χ^2^ test. For the multiple comparisons, a Bonferroni correction was applied. To determine factors associated with the prelaminar tissue thickness in OAG patients, multivariate linear regression analyses were used. The dependent variable was the prelaminar tissue thickness. For multiple linear regression analyses, variables with P < 0.15 (presence of type 2 DM, disc area, and mean deviation) in univariate analyses were included in the multivariate model. A value of *P* < 0.05 was considered statistically significant.

## Results

A total of 333 patients with diabetes received an ophthalmic examination between July 2014 and July 2015. Among them, 134 patients having normal optic disc with or without RNFL defect were excluded. Among them, 80 were excluded due to absence of glaucomatous VF defect, 30 were excluded due to presence of PDR or macula edema, and 25 were excluded because of poor EDI images. Finally, 64 OAG+DM patients, 68 age-matched OAG-DM patients, and 36 healthy control subjects were included in this study. There were no statistically significant differences in age, SE, IOP, and central corneal thickness (CCT) among the groups (*P* = 0.806, 0.486, 0.185, and 0.275, respectively). The average duration of diabetes in the OAG+DM group was 11.3 ± 8.2 years. The OAG+DM group included 39 (60.9%) patients with no DR, 16 (25.0%) with mild NPDR, and 9 (14.1%) with moderate NPDR. No patients in this group had PDR. The interobserver ICC for measuring the prelaminar tissue thickness, laminar cribrosa depth and laminar cribrosa thickness were 0.894 (95% confidence interval (CI): 0.725, 0.959), 0.827 (95% CI, 0.573, 0.930), and 873 (95% CI, 0.670, 0.951), respectively. And the intraobserver ICC for measuring the prelaminar tissue thickness, laminar cribrosa depth and laminar cribrosa thickness were 0.910 (95% CI, 0.846, 0.948), 0.995 (95% CI, 0.993, 0.996), and 0.962 (95% CI, 0.934, 0.978), respectively.

Mean deviation and average RNFL thicknesses measured using OCT were significantly different among the groups (both, *P* < 0.001, Table 1). In *post hoc* tests, the control group had significantly higher mean deviation and average RNFL thicknesses than the OAG+DM and OAG-DM groups (both, *P* < 0.001). However, there were no significant differences in either measurement between the OAG+DM and OAG-DM groups (both, *P* > 0.999).

**Table 1:** Demographics of open angle glaucoma patients and age-matched control subjects.

	OAG		Age-matched controls	*P* value	*Post hoc* testing
	with type 2 diabetes	without type 2 diabetes			
	n = 64	n = 68	n = 36		
Age, years	59.4 ±12.3 (35~81)	59.4 ± 13.2 (33–80)	57.9 ± 9.4 (43–70)	0.806	
Spherical Equivalent, D	-1.08 ± 2.18 (-7.0~+1.88)	-0.71 ± 2.13 (-7.88~+2.63)	-1.30 ± 0.51 (-7.88~+2.60)	0.486	
CCT, μm	556.3 ± 32.6 (454~614)	544.1 ± 47.3 (453~654)	551.5 ± 30.9 (485~596)	0.275	
IOP, mmHg	14.5 ± 3.5 (8~26)	13.5 ± 3.7 (8~22)	13.4 ± 3.0 (8~20)	0.185	
Lens status					
No cataract, n (%)	41 (64.1%)	45 66.2%)	23 (63.9%)	0.895	
Mild cataract, n (%)	16 (25.0%)	13 (19.1%)	9 (25.0%)		
Pseudophakia, n (%)	7 (10.9%)	10 (14.7%)	4 (11.1%)		
Anti-glaucoma medication					
PGA, n (%)	23 (35.9)	42 (61.7)	NA	0.003	
Beta-blocker, n (%)	37 (57.8)	39 (57.3)	NA	0.549	
Topical carbonic anhydrase inhibitor, n (%)	23 (35.9)	26 (38.2)	NA	0.463	
Alpha-agonist, n (%)	17 (26.6)	15 (22.1)	NA	0.344	
Pilocarpine, n (%)	1 (1.5)	1 (1.5)	NA	0.737	
Total no. of anti-glaucoma medication			NA		
0	5 (7.8)	0 (0)	NA	0.001	
1	54 (84.4)	49 (72.1)	NA		
≥2	5 (7.8)	19 (27.9)	NA		
Mean deviation, dB	-4.3 ± 3.9 (-20.93~+0.22)	-5.1 ± 3.2 (-13.81~+0.04)	-0.8 ± 1.4 (-3.01~+2.56)	<0.001	OAG+DM< C, OAG-DM<C
Disc area, mm^2^	2.19 ± 0.57 (0.64~3.58)	2.10 ± 0.42 (1.12~3.21)	2.10 ± 0.42 (1.36~3.22)	0.477	
Rim area, mm^2^	0.95 ± 0.23 (0.46~1.47)	0.95 ± 0.27 (0.52~2.37)	1.20 ± 0.26 (0.70~1.83)	<0.001	OAG+DM< C, OAG-DM<C
Vertical cup-to disc ratio	0.72 ± 0.08 (0.45~0.86)	0.74 ± 0.10 (0.46~0.92)	0.58 ± 0.15 (0.40~0.83)	<0.001	OAG+DM< C, OAG-DM<C
Average RNFL thickness, μm	78.8 ± 12.0 (50~107)	77.9 ± 11.5 (54~106)	92.7 ± 9.2 (76~115)	<0.001	OAG+DM< C, OAG-DM<C

OAG, open-angle glaucoma; D, diopters; IOP, intraocular pressure; CCT, central corneal thickness; PGA; prostaglandin analogue, RNFL, retinal nerve fiber layer.

Table 2 shows the differences in the locations of VF defects in OAG patients with or without DM. There were 46 (71.9%) and 47 (69.1%) isolated hemifield defects in the OAG+DM and OAG-DM groups, respectively (*P* = 0.849; χ^2^ test). The defects in the OAG+DM group were most commonly found in the inferior hemifield, whereas those in the OAG-DM group tended to occur in the superior hemifield (Bonferroni-adjusted *P* = 0.030; χ^2^ test). In addition, the OAG+DM group tended to involve the central VF (*P* = 0.044; χ^2^ test).

**Table 2:** Location of visual field defect in open angle glaucoma patients with or without type 2 diabetes.

	OAG with type 2 diabetes	OAG without type 2 diabetes	*P* value
According to the vertical location of scotoma			
Superior, n (%)	18 (28.1)	33 (48.5)	0.010*
Inferior, n (%)	28 (41.2)	14 (20.6)	
Both, n (%)	18 (28.1)	21 (30.9)	
According to the involvement of central visual field			
With central visual field involvement, n (%)	29 (45.3)	20 (29.4)	0.044
Without central visual field involvement, n (%)	35 (54.7)	48 (70.6)	

OAG, open-angle glaucoma.

* Bonferroni-adjusted P = 0.030

**Fig 2:**
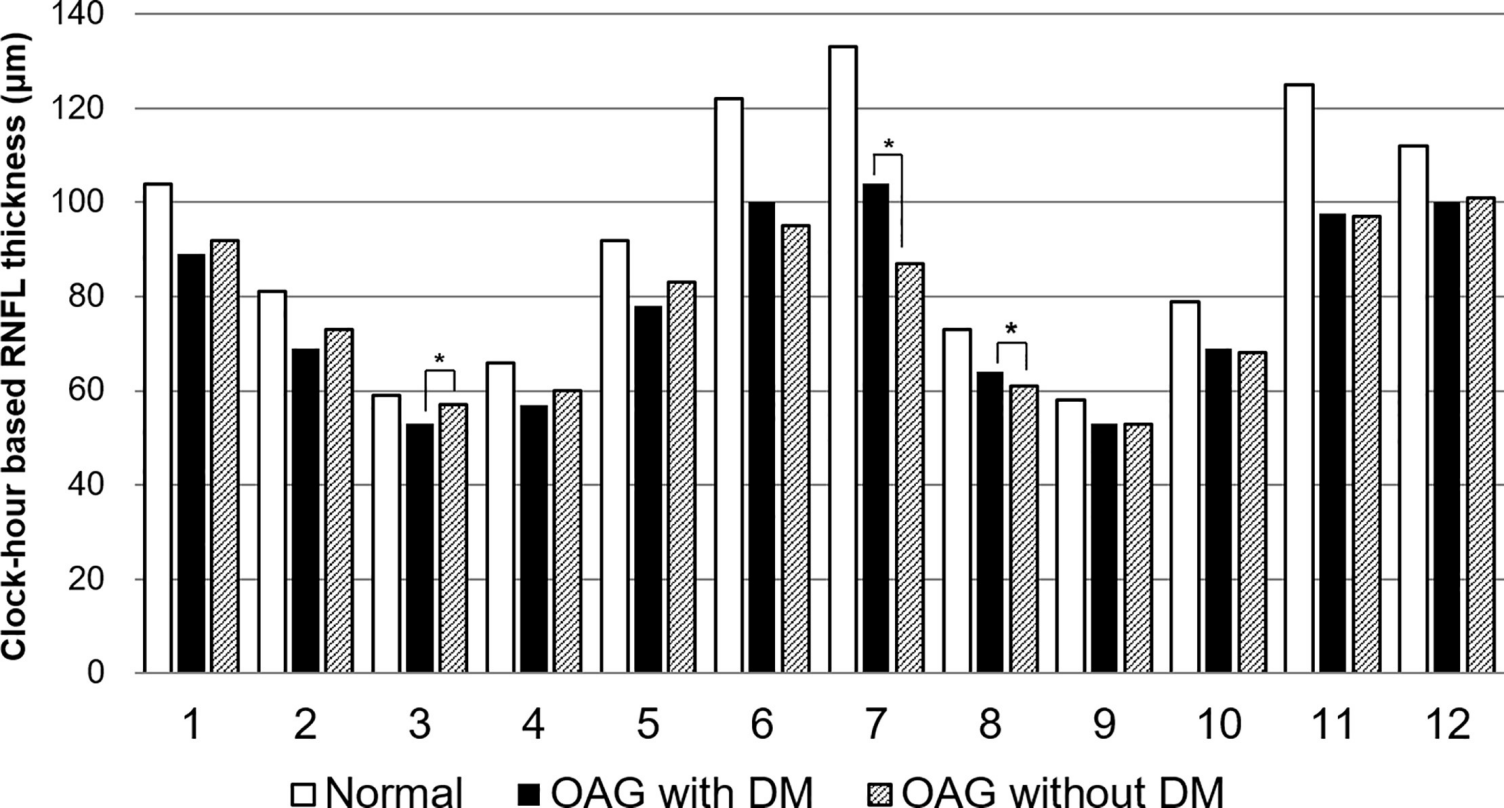
Clock-hour-based retinal nerve fiber layer thicknesses in open-angle glaucoma patients with or without type 2 diabetes and normal controls. In comparisons of the clock-hour retinal nerve fiber layer (RNFL) thicknesses among normal controls and OAG patients with or without DM, significant differences of RNFL thicknesses were noted among groups in every clock-hour segment except 9 o’clock side (*P* < 0.05 in 1–8,10–12 o’clock side, *P* = 0.074 at 9 o’clock side, ANOVA). In *post-hoc* testing, the OAG+DM group tended to have a relatively thinner RNFL on the superior side of the ONH (*P* = 0.030 at the 3 o’clock side, asterix), whereas the OAG-DM group showed a relatively thinner RNFL on the inferior side (*P* < 0.001, 0.027 at the 7 and 8 o’clock sides, respectively, asterix).

In comparisons of the clock-hour RNFL thicknesses between OAG patients with or without DM, the OAG+DM group tended to have a relatively thinner RNFL on the superior side of the ONH (*P* = 0.016 at the 3 o’clock side), whereas the OAG-DM group showed a relatively thinner RNFL on the inferior side (*P* < 0.001, 0.027 at the 7 and 8 o’clock sides, respectively; Fig 2).

**Fig 3:**
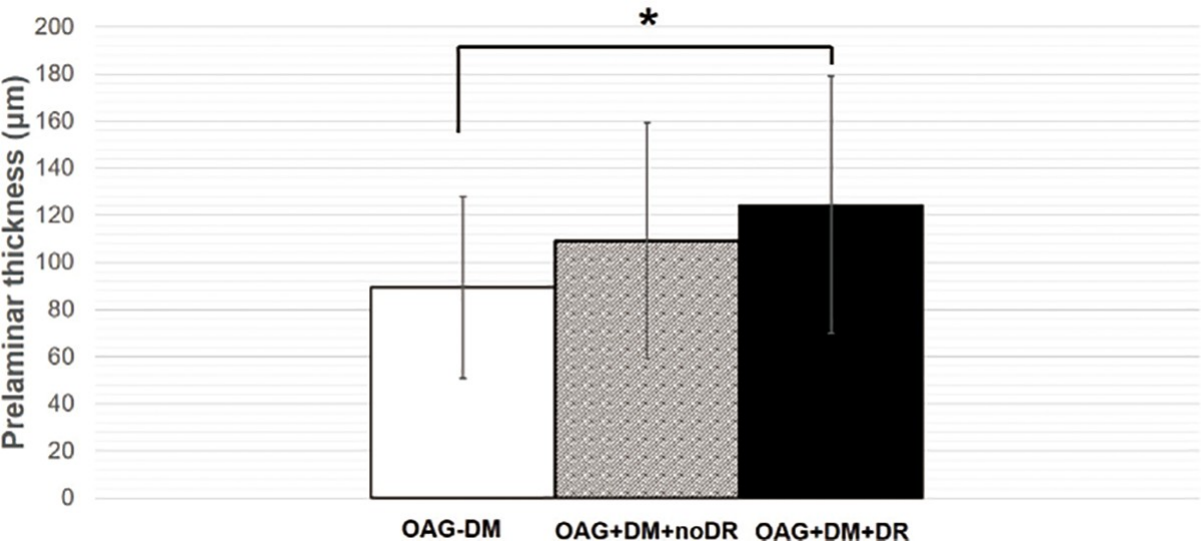
Comparison of the prelaminar thickness among open-angle glaucoma patients. Among open angle glaucoma (OAG) patients, there were gradual prelaminar thickening in the order of the OAG-DM patients, the OAG+DM patients without diabetic retinopathy (DR), and the OAG+DM patients with DR (P = 0.010) In *post hoc* testing, the OAG+DM patients with DR had significantly higher thicker prelaminar tissue than the OAG-DM patients (*P =* 0.015*)*.

Among the groups, the prelaminar tissue thickness was highest in the OAG+DM group, followed by the control subjects and the OAG-DM group (*P* = 0.035; Table 3). In *post hoc* tests, the prelaminar tissue thickness was significantly higher in the OAG+DM group than in the OAG-DM group (*P* = 0.033). Among OAG patients, there was gradual prelaminar thickening in the order of the OAG-DM patients, the OAG+DM patients without DR, and the OAG+DM patients with DR (P = 0.010, Fig 3). In *post hoc* tests, the OAG+DM patients with DR had significantly higher thicker prelaminar tissue than the OAG-DM patients (*P =* 0.015*)* The lamina cribrosa depth was deepest in the OAG+DM group followed by the OAG-DM group and controls (*P* = 0.006), being significantly deeper than that in controls (*P* = 0.007) but without significance between the other groups (*P* = 0.074). No significant differences were noted in the lamina cribrosa thickness (*P* = 0.507) Macular choroidal thickness was significantly thinner in the OAG+DM group and OAG-DM group compared with controls (P<0.001). In both univariate and multivariate analyses, the presence of DM was a significant factor affecting the prelaminar tissue thickness in OAG patients (Table 4).

**Table 3:** Optic nerve head parmaeters and macular choroidal thickness in open angle glaucoma patients with or without type 2 diabetes and age-matched controls.

	OAG		Age-matched controls	*P* value	*Post hoc* testing		
	with type 2 diabetes	without type 2 diabetes			With vs without DM	With DM vs. control	Without DM vs. control
ONH parameters							
Prelaminar thickness, μm	115.3 ± 51.9	89.4 ± 38.6	105.2 ± 68.5	0.035	0.033	0.999	0.581
Anterior lamina depth, μm	790.3 ± 163.6	714.8 ± 169.4	657.6 ± 168.6	0.006	0.074	0.007	0.477
Lamina cribrosa thickness, μm	226.8 ±73.2	235.7 ± 72.0	249.3 ± 61.6	0.507	0.999	0.748	0.999
Macular choroidal thickness							
Subfoveal choroidal thickness, μm	103.0 ± 24.5	98.4 ± 23.1	247.6 ± 56.9	<0.001	0.999	<0.001	<0.001
Temporal macular choroidal thickness, μm	90.9 ± 18.0	85.8 ± 17.5	255.0 ± 58.5	<0.001	0.999	<0.001	<0.001
Nasal side macular choroidal thickness, μm	85.0 ± 17.9	85.8 ± 22.0	242.9 ± 54.3	<0.001	0.999	<0.001	<0.001

DM, diabetes mellitus; ONH, optic nerve head

**Table 4:** Univariate and multivariate regression analysis: Variables associated with prelaminar thickness in open angle glaucoma patients.

	Univariate analysis			Multivariate analysis		
Parameters	B	99% CI	P value	B	99% CI	P value
Presence of type 2 diabetes	27.39	8.91,45.86	0.004	20.19	63.58, .36.81	0.018
Age	0.39	-0.33, -1.12	0.284			
Disc area	11.35	-7.14, 29.84	0.226			
Spherical equivalent	-2.16	-6.48, 2.14	0.321			
CCT	0.10	-0.15, 0.36	0.415			
Average RNFL thickness	-0.53	-1.31, 0.25	0.180	-0.56	-1.32, 0,21	0.152

CI, confidence interval; CCT, central corneal thickness; RNFL, retinal nerve fiber layer.

## Discussion

OAG patients with type 2 diabetes tended to have a glaucomatous VF defect in the inferior hemifield, and glaucomatous RNFL damage at the superior side of the ONH (Tables 2 and 3). Our results are consistent with previous studies that have found that OAG patients with inferior VF defects have a significantly higher frequency of diabetes[26] and vice versa.[27]

RNFL loss, which is common in patients with diabetes as a manifestation of non-glaucomatous diabetic optic neuropathy, occurs predominantly in the superior hemisphere.[13, 14, 28, 29] This is usually related to vascular insufficiency associated with diabetes, which is the key pathogenic mechanism of DR.[12, 29, 30] However, the key pathogenic feature of glaucoma is mechanical deformation of the lamina cribrosa, such as posterior displacement, thinning, and compression of the lamina cribrosa, and the inferior temporal side of the lamina cribrosa is more susceptible to damage from nerve fibers that pass through the region (Fig 2; Table 2). Posterior displacement of the lamina cribrosa and the resultant deepening of the lamina cribrosa are important structural parameters of glaucomatous changes, because they occur mostly in very early-stage glaucoma,[31] and ONH surface changes have been shown to precede changes in the RNFL.[32] Notably, both diabetic and non-diabetic OAG patients had deeper lamina cribrosa than control subjects. However, they also exhibited a different pattern of glaucomatous damage from non-diabetic OAG patients, although they were both accompanied by a comparable degree of mechanical deformation. Lee *et al*. reported that changes in retinal capillary blood flow were more closely related to ONH morphology in diabetic OAG patients than in non-diabetic OAG patients, which suggests that retinal blood flow insufficiency plays a larger role in glaucomatous ONH progression in diabetics’ eyes.[33] Taken together, these findings suggest that additional pathogenic factors may play a role in the pathogenesis of OAG in patients with diabetes.

The prelaminar tissue is located just above the lamina cribrosa, and is composed of neuronal and glial tissue. With the progression of glaucoma, the thicknesses of the prelaminar tissue and lamina cribrosa decrease.[24, 34] However, unlike the lamina cribrosa, the prelaminar tissue is characterized by its plasticity, and quickly responds to changes in IOP. A rise in IOP results in compression of the prelaminar tissue,[35] which then thickens after surgical IOP reduction.[36]

Consistent with previous studies, in our study, the thickness of the prelaminar tissue was lowest in non-diabetic OAG patients (Table 3).[34] However, we found paradoxical thickening of the prelaminar tissue in eyes with diabetic OAG. Such thickening can be caused by an increased glial reaction, connective tissue remodeling, or blockage of axonal transport,[37] considering that there were no difference in macular choroidal thickness between diabetic and non-diabetic OAG patients (Table 3). In diabetes, the biomechanical properties of the ONH are altered. Terai *et al*.[15] reported that increased stiffness was noted in the ONH and peripapillary sclera in animal models, which could be caused by an accumulation of advanced end products and high glucose levels in diabetic patients.[16] Retrograde axonal transport is also impaired in the ONH of rats with streptozotocin-induced diabetes.[38, 39] This sclerotic change in the ONH in diabetes may affect the axonal transport and result in the thickening of the prelaminar tissue. In addition, in diabetes, hyperglycemia and inflammation affect retinal glial cells, resulting in increased glial reactivation, particularly in the early course of the disease.[40] In the future, a longitudinal study is required to determine the significance of the role of prelaminar tissue thickening on the progression of glaucoma in diabetic patients.

In our study, the laminar depth was deepest in the diabetic OAG patients, followed by the non-diabetic OAG patients and the controls (Table 3). However, Yokota et al. who compared the structure of the lamina cribrosa between eyes with and without neovascular glaucoma, reported that the depth and thickness of the lamina cribrosa were not different. It is inferred that the effects of IOP on the structure of lamina cribrosa depends on the nature of IOP elevation. Short-term IOP elevation did not evoke significant changes in the structure of the lamina cribrosa.[41] Beotra et al. also found that subjects with angle closure glaucoma experience lower strains in lamina cribrosa than those with open angle glaucoma.[42] In addition, there was no statistically significant difference in the lamina depth between non-diabetic OAG patients and the controls on post-hoc testing (Table 3). This result seems to be associated with the relatively high proportion of normal tension glaucoma in Korean population, occupying 77% of the open angle glaucoma.[43] Li et al. has shown that the lamina cribrosa is more posteriorly located in high tension glaucoma than normal tension glaucoma, and the parameter does not have a good level of diagnostic accuracy for detecting normal tension glaucoma.[44] Furthermore, the IOP was highest in diabetic OAG patients, followed by non-diabetic OAG patients in our study. Akkaya et al.[45] showed that diabetic patients without glaucoma showed thicker and more anteriorly positioned lamina cribrosa compared with those for healthy controls, suggesting that the neuroprotective effect of diabetes is related with those structural characteristics. In the ocular hypertension treatment study,(6) diabetes exhibited protective effects on the development of glaucoma. However, in advanced glaucoma intervention study,[46] diabetes was shown to be one of the risk factors for glaucoma progression. In this regards, it seems that the effects of diabetes on the ONH are different according to the stage of glaucoma. Further studies on the transition of laminar position and thickness according to glaucoma progression in diabetic patients are necessary.

The pattern of the VF impacts vision-related quality of life. A superior hemifield defect is strongly associated with difficulty with near activity, whereas an inferior hemifield defect impacts vision-specific role difficulties and general and peripheral vision.[47] Clusters in the inferior paracental VF of healthy eyes are particularly strongly associated with the quality of life.[48] In the present study, OAG patients with diabetes tended to have an inferior VF defect. As their diabetes progressed, they were likely to receive further interventions, such as panretinal photocoagulation or intravitreal injection, which additionally affected their visual functions. Therefore, personalized advice should be provided to OAG patients with diabetes, accompanied by a more detailed medical history review in these patients.

There were some potential limitations in this study. First, we excluded eyes with PDR or neovascular glaucoma, because the reliability of OCT images for these disorders was low. Thus, OAG patients with advanced DR may have different characteristics than shown in this study. Second, the results should be interpreted in the context of the study design. A major limitation is the retrospective nature of data collection. In addition, this study is clinically based and did not use population-based screening. Third, we measured only the central three points in each B-scan, which precisely showed the structural characteristics of the lamina cribrosa, and we only measured the prelaminar tissue thickness and lamina cribrosa depth among other ONH parameters, because the precise measurement of ONH was not the primary goal of our study. In addition, the lateral magnification was not accounted for in the OCT imaging in this study. The lateral magnification may affect the measurement in lamina cribrosa configuration, especially in case of large eyeballs. Although all subjects were required to have a limited range of spherical refraction and cylinder correction in this study, this could influence the results, considering the wide range of refractive errors and hence axial lengths included. The main aim of this study is to investigate the ONH characteristics in glaucoma patients with diabetes. Regarding the comparison of VF defect patterns between groups, we chose the post-hoc tests, instead of the planned contrasts. Because this is a post hoc exploratory finding, the results must be interpreted in light of the restrictions inherent in the study design. Finally, conditions such as lens opacities could have affected the enhanced depth imaging images, particularly in patients with diabetes, so we discarded cases with poor enhanced depth imaging OCT images and utilized Cirrus OCT scans with signal strengths ≥ 6.

In summary, diabetic OAG patients exhibited different characteristics of glaucoma than non-diabetic OAG patients. Importantly, significant thickening of the prelaminar tissue was found in diabetic OAG patients. Overall, our results indicate that the pathogenic mechanism of glaucoma may be altered by diabetes, and thus OAG patients with diabetes need to be customized in clinical practice.

## Supporting information

S1 Table Baseline and structural data in open-angle glaucoma patients with or without type 2 diabetes and normal controls. (XLS)
